# Design and development of innovative microparticulate/nanoparticulate inhalable dry powders of a novel synthetic trifluorinated chalcone derivative and Nrf2 agonist

**DOI:** 10.1038/s41598-020-76585-2

**Published:** 2020-11-13

**Authors:** Priya Muralidharan, Brielle Jones, Graham Allaway, Shyam S. Biswal, Heidi M. Mansour

**Affiliations:** 1 Colleges of Pharmacy and Medicine, University of Arizona, 1703 E. Mabel St, Tucson, AZ 85721 USA; 2 Cureveda LLC, Halethorpe, MD USA; 3 Department of Environmental Health Sciences, Bloomberg School of Public Health, Johns Hopkins University, Baltimore, MD USA; 4 Division of Pulmonary and Critical Care Medicine, School of Medicine, Johns Hopkins University, Baltimore, MD USA; 5 Division of Translational and Regenerative Medicine, Department of Medicine, The University of Arizona College of Medicine, Tucson, AZ USA; 6 The BIO5 Research Institute, The University of Arizona, Tucson, AZ USA; 7 Institute of the Environment, The University of Arizona, Tucson, AZ USA; 8 National Cancer Institute Comprehensive Cancer Center, The University of Arizona, Tucson, AZ USA

**Keywords:** Drug development, Preclinical research, Translational research, Drug delivery, Drug delivery, Pharmaceutics, Drug discovery, Nanoscience and technology

## Abstract

Chalcone derivatives are shown to possess excellent anti-inflammatory and anti-oxidant properties which are of great interest in treating respiratory diseases such as acute lung injury (ALI), acute respiratory distress syndrome (ARDS), chronic obstructive pulmonary disease (COPD), and pulmonary fibrosis (PF). This study successfully designed and developed dry powder inhaler (DPI) formulations of TMC (2-trifluoromethyl-2′-methoxychalone), a new synthetic trifluorinated chalcone and Nrf2 agonist, for targeted pulmonary inhalation aerosol drug delivery. An advanced co-spray drying particle engineering technique was used to design and produce microparticulate/nanoparticulate formulations of TMC with a suitable excipient (mannitol) as inhalable particles with tailored particle properties for inhalation. Raw TMC and co-spray dried TMC formulations were comprehensively characterized for the first time using scanning electron microscopy (SEM) with energy dispersive X-ray (EDX) spectroscopy, thermal analysis, X-ray powder diffraction (XRPD), and molecular fingerprinting as dry powders by ATR-FTIR spectroscopy and Raman spectroscopy. Further, biocompatibility and suitability of formulations were tested with in vitro cellular transepithelial electrical resistance (TEER) in air-interface culture (AIC) using a human pulmonary airway cell line. The ability of these TMC formulations to perform as aerosolized dry powders was systematically evaluated by design of experiments (DOEs) using three different FDA-approved human inhaler devices followed by interaction parameter analyses. Multiple spray drying pump rates (25%, 75%, and 100%) successfully produced co-spray dried TMC:mannitol powders. Raw TMC exhibited a first-order phase transition temperature at 58.15 ± 0.38 °C. Furthermore, the results demonstrate that these innovative TMC dry powder particles are suitable for targeted delivery to the airways by inhalation.

## Introduction

Airways are continuously exposed to environmental insults that trigger inflammation consistent with the body’s natural response to a stimulus such as smoke, infection, pollutants, and allergens. Hence, inflammation is the main characteristic of many respiratory diseases such as pneumonia, acute lung injury (ALI)^1^, acute respiratory distress syndrome (ARDS), asthma, chronic obstructive pulmonary disease (COPD)^2^, and pulmonary fibrosis (PF)^3,4^. The innate defense mechanism of the respiratory system includes mechanical barrier, mucus production, and ciliary movement to remove the pathogen, phagocytosis and inflammatory response. Pulmonary inflammation can be acute, as seen in ALI and ARDS, or it can be chronic such as in asthma and COPD. In acute lung inflammation, the body fights the trigger immediately to resolve and restore homeostasis, if this fails it leads to chronic inflammation^5^. The inflammatory cells involved in acute and chronic inflammation are different. Neutrophils, eosinophils, are more active in acute cases whereas lymphocytes and macrophages play a dominant role in chronic inflammation^5,6^. There are several cellular and molecular mechanisms involved in the repair and resolution of lung inflammation^6^, among those the anti-oxidant signaling pathways are of growing interest. Nuclear factor (erythroid-derived 2)—like 2 (Nrf2) genetic pathway is an anti-oxidant signaling pathway that controls the anti-oxidant and anti-inflammatory response against oxidative stress. In many lung diseases, there is an inherent risk for oxidative stress due to the high oxygen burden in the organ, as reported in chronic obstructive pulmonary disease (COPD) and pulmonary hypertension^7–11^.

**Figure 1:**
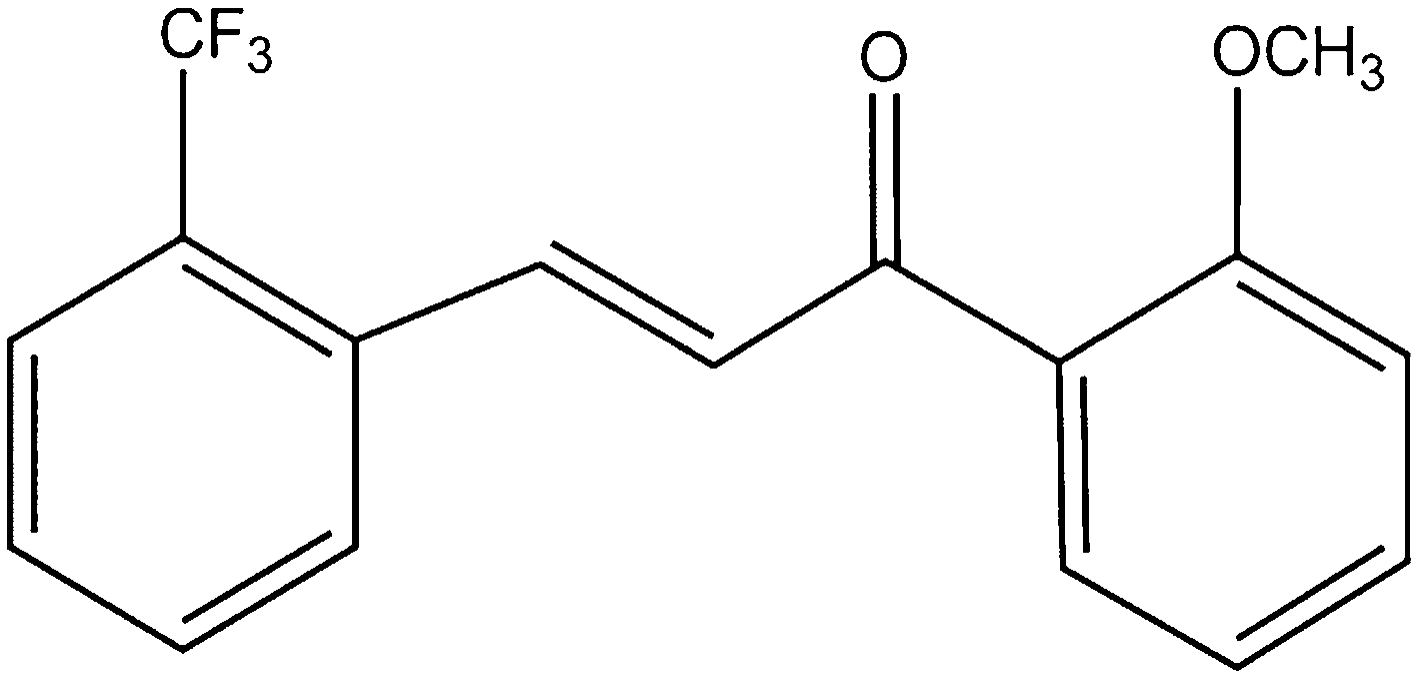
Chemical structure of TMC.

Recently, the involvement of Nrf2 in pulmonary diseases such as COPD, ALI^1^, and PF and targeting with Nrf2 activators are of great interest^12–20^. The Nrf2 activity of a novel synthetic trifluorinated chalcone chemical derivative, TMC (2-trifluoromethyl-2′-methoxychalone), was reported^21^. The study synthesized about 59 derivatives of chalcone and systematically screened their Nrf2 activity by testing in normal pulmonary epithelial cell line and mouse model. Among the synthesized derivatives, TMC (the chemical structure is shown in Fig. 1) emerged as a potential lead compound possessing Nrf2 pathway activator properties. Chalcone is also known as 1,3-diaryl-2-propen-1-ones is an aromatic ketone that exists as either cis (*Z*) or trans (*E*) isomeric form. It belongs to the flavonoid family naturally occurring in fruits, vegetables, and other plants. There are numerous derivatives of chalcone studied for their biological activities as anti-inflammatory^22–24^, anti-histaminic^25^, anti-oxidant^26,27^, anti-cancer^28–31^, antituberculosis^32^, nitric oxide inhibitor^33^, and antifungal^34^. The anti-inflammatory effect of chalcones is demonstrated through the NF-κB pathway inhibition^35–37^. Also reported is the Nrf2/ARE cellular pathway activation efficacy of chalcone and its derivatives in different cell lines such as mouse embryonic fibroblast cells^38^, neural and microglial cells^39,40^, AREc32 cells^41^, gastric epithelial cells^42^, lung epithelial cells^21^, and colon cells^43^. More importantly, the Nrf2 activation and anti-fibrotic effects of a chalcone molecule were recently demonstrated in in vitro human skin fibroblasts and in an in vivo skin fibrosis mouse model^44^.

The main objective of this systematic study was to design, develop and optimize dry powder inhaler (DPI) formulations of TMC (Fig. 1) to treat inflammatory and fibrotic lung diseases directly through targeted lung delivery to the airways. To the authors’ knowledge, this study is the first to report on the solid-state physicochemical properties of TMC and successfully create innovative TMC inhalable nanoparticles/microparticles. TMC was co-spray dried with mannitol as an excipient. Table 1 lists the powder formulations that were created in this study. The aerosol formulations were comprehensively characterized and examined in vitro for its suitability as pulmonary drug delivery. The influence of different human DPI devices on the aerosol properties of the formulations was also quantified and correlated with DPI device properties and formulation properties.

**Table 1:** Advanced spray drying parameters for co-spray dried (co-SD) powders from methanol (MeOH) solution using organic solution advanced closed mode spray drying particle engineering design.

Powder composition (molar ratio)	Molar ratio composition (TMC:Man)	Feed concentration in MeOH (% w/v)	Pump rate (%)	Inlet T (°C)	Outlet T (°C)
Co-SD TMC:Man	25:75	0.156	25	89–90	50–51
Co-SD TMC:Man	25:75	0.156	50*	90	40–44
Co-SD TMC:Man	25:75	0.156	75	90	29–36
Co-SD TMC:Man	25:75	0.156	100	90–91	18–23

*Not used for further experiments due to insufficient mass.

## Results

### Scanning electron microscopy (SEM) and energy dispersive X-ray (EDX)

**Figure 2:**
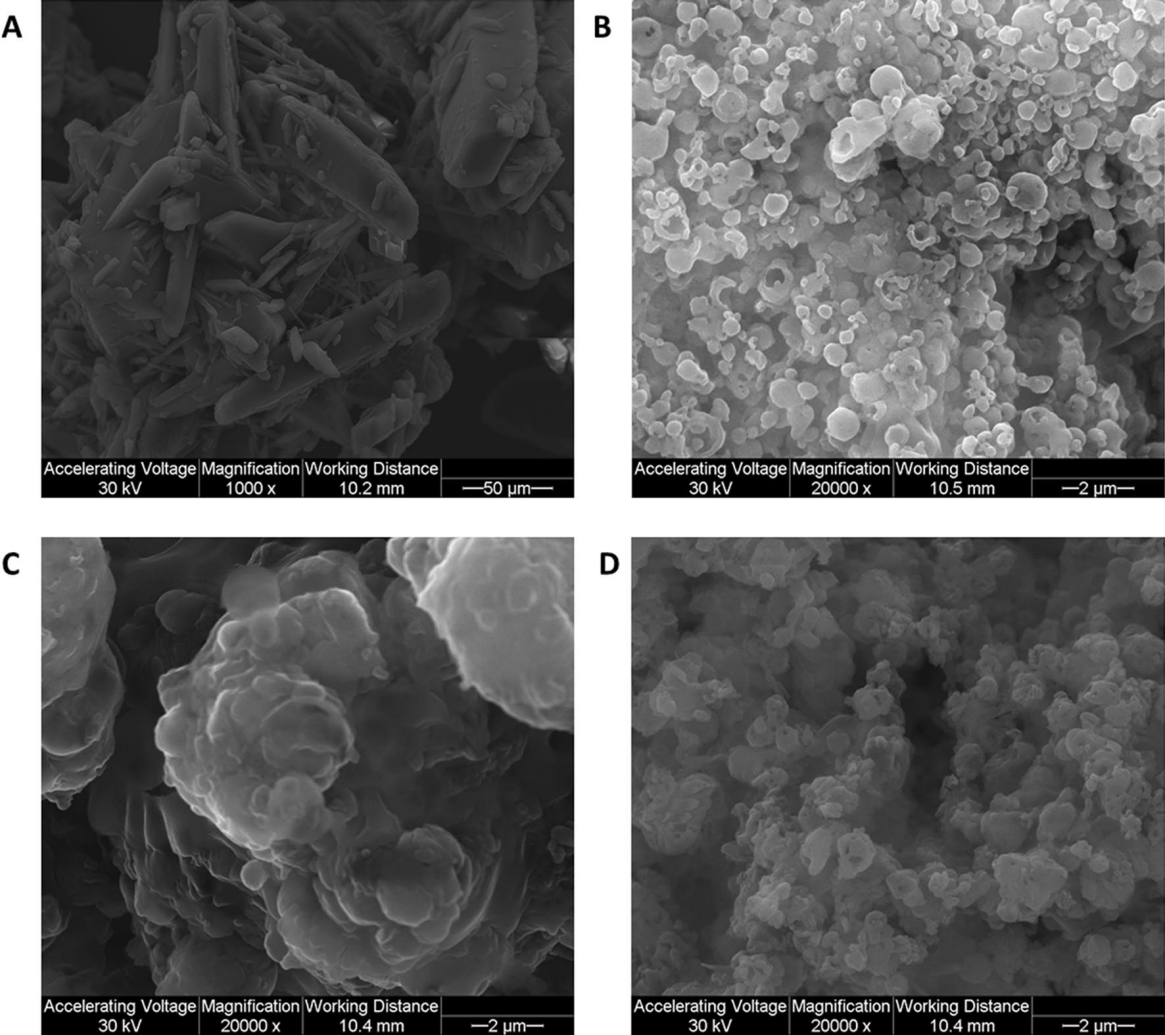
Scanning electron microscopy (SEM) micrographs of (**A**) Raw TMC at × 1000; (**B**) co-SD TMC: Man at 25% PR; (**C**) co-SD TMC: Man at 75% PR; (**D**) co-SD TMC: Man at 100% PR. (**B**–**D**) Images are at × 20,000 magnification.

Figure 2 displays the scanning electron micrograph of raw TMC and co-SD TMC: Man particles at different spray drying pump rates (PRs). The raw TMC particles were elongated and exhibited a wide size range with sizes much too large for inhalation aerosol delivery to the lungs. Particle engineering design by advanced co-spray drying TMC with Man successfully rendered the particles into small and equivalent spheres which are essential solid-state particle properties for DPIs. The particles made at 25% PR had smooth surface morphology. Similarly, 100% PR particles had smooth surface morphology with equivalent spheres. The particles SD at 75% PR had a different appearance compared to that of 25% and 100% PR particles. The particles were large where more solid sintering was observed.

**Figure 3:**
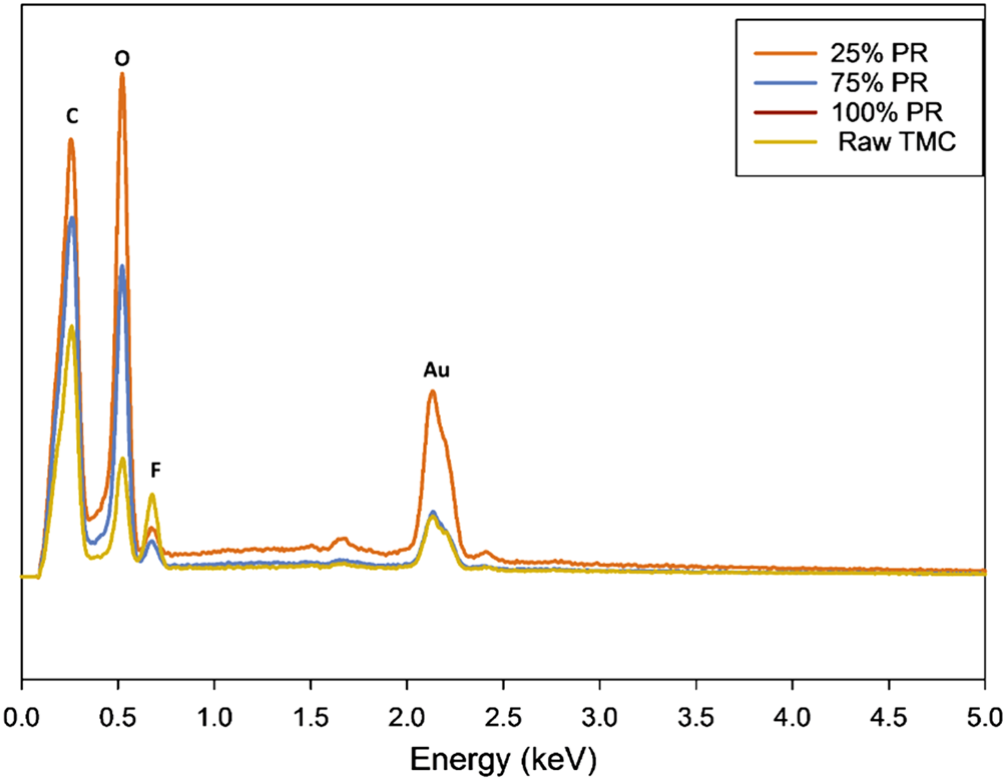
Energy dispersive spectrum (EDX) of Raw TMC and co-SD TMC: Man particles.

The EDX spectra of the raw and co-SD particles are displayed in Fig. 3. The elemental analysis of raw TMC and co-SD TMC: Man particles show peaks of carbon, oxygen, fluorine, and gold at 0.269, 0.520, 0.678, and 2.148 respectively. The presence of fluorine at 0.678 keV energy is elemental atomic evidence in the solid-state of the presence of TMC molecules in the co-SD particles at all three spray drying pump rates.

### Particle sizing and size distribution by image analysis of SEM micrographs

The SEM micrographs show discrete particles of co-SD TMC: Man which were measured using imaging software SigmaScan Pro version 5.0 (Systat software, San Jose, CA). The sizing data is shown in Table 2. The average particle size of 25% PR, 75% PR and 100% PR were 0.746 µm, 1.37 µm, and 0.962 µm, respectively. The number based diameters measured were D_n10_ = 0.52 µm, 0.79 µm, and 0.60 µm for 25%, 75% and 100% PR, respectively, and D_n90_ = 0.98 µm, 2.26 µm, and 1.40 µm for 25%, 75% and 100% PR, respectively.

**Table 2:** Particle sizing using image analysis on SEM micrographs (n ≥ 100 particles).

Powder composition (molar ratio)	Spray drying pump rate (%)	D_n10_ (µm)	D_n90_ (µm)	Average size (µm)	Span
Co-SD TMC:Man 25:75	25	0.52	0.98	0.74	0.63
Co-SD TMC:Man 25:75	75	0.79	2.26	1.37	1.27
Co-SD TMC:Man 25:75	100	0.60	1.40	0.96	0.96

### X-ray powder diffraction (XRPD)

**Figure 4:**
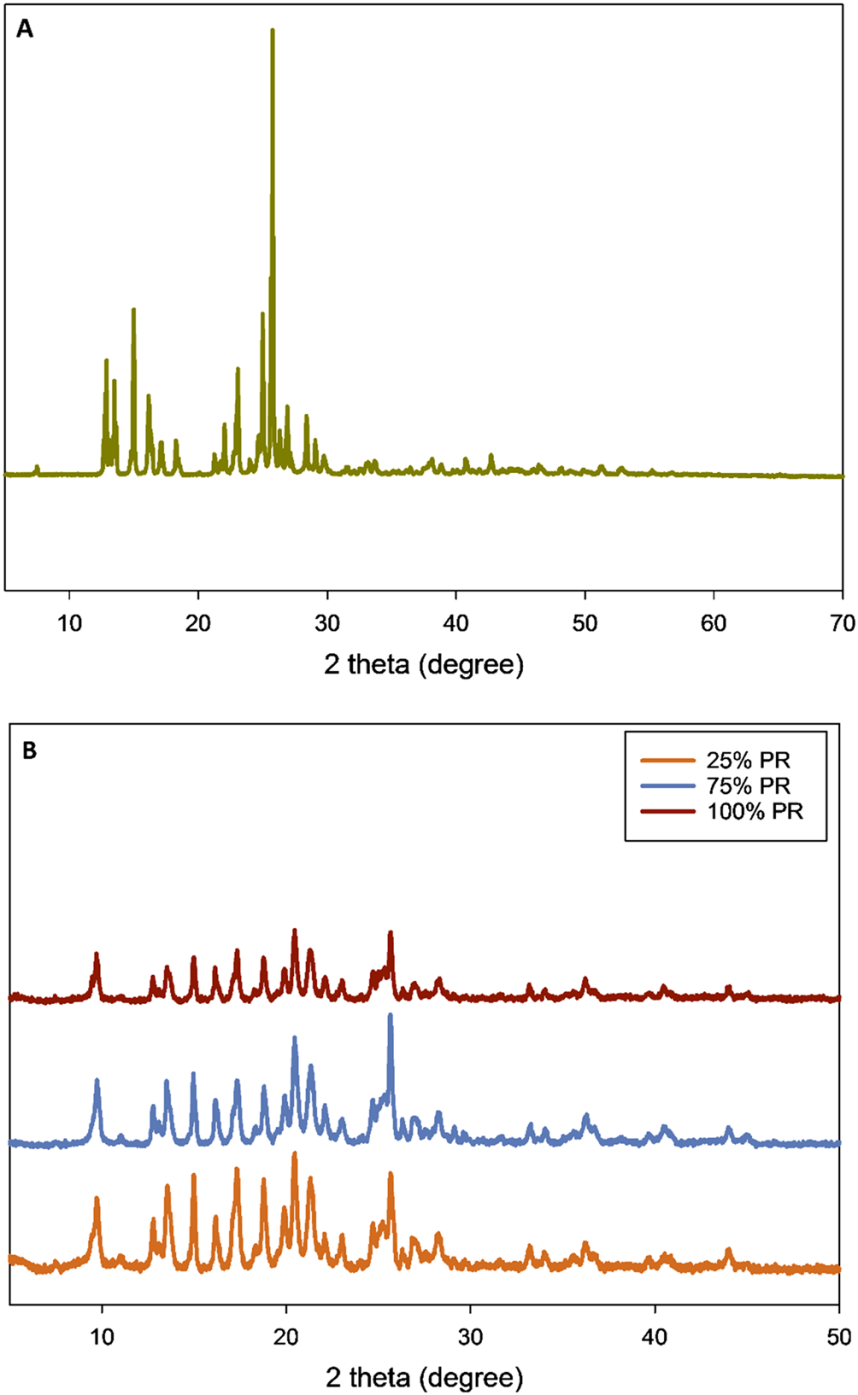
X-ray powder diffraction (XRPD) patterns of (**A**) Raw TMC; and (**B**) co-SD TMC: Man particles.

The X-ray diffraction of raw TMC shows several sharp and intense peaks, as evident from Fig. 4A. This is indicative of long-range molecular order as a result of crystallinity in the solid state. Prominent peaks of crystalline TMC were seen at 12.8, 13.5, 14.9, 16.1, 17.1, 18.3, 22.0, 23.0, 24.9, 25.7, 26.9 and 28.4 2θ degrees. The co-SD TMC: Man particles also showed several sharp and intense peaks, as seen in Fig. 4B, indicative of crystallinity and the peaks correspond to those of both components. It has been previously reported that d-mannitol retains its crystallinity following advanced spray drying under our conditions^45^.

### Differential scanning calorimetry (DSC)

**Figure 5:**
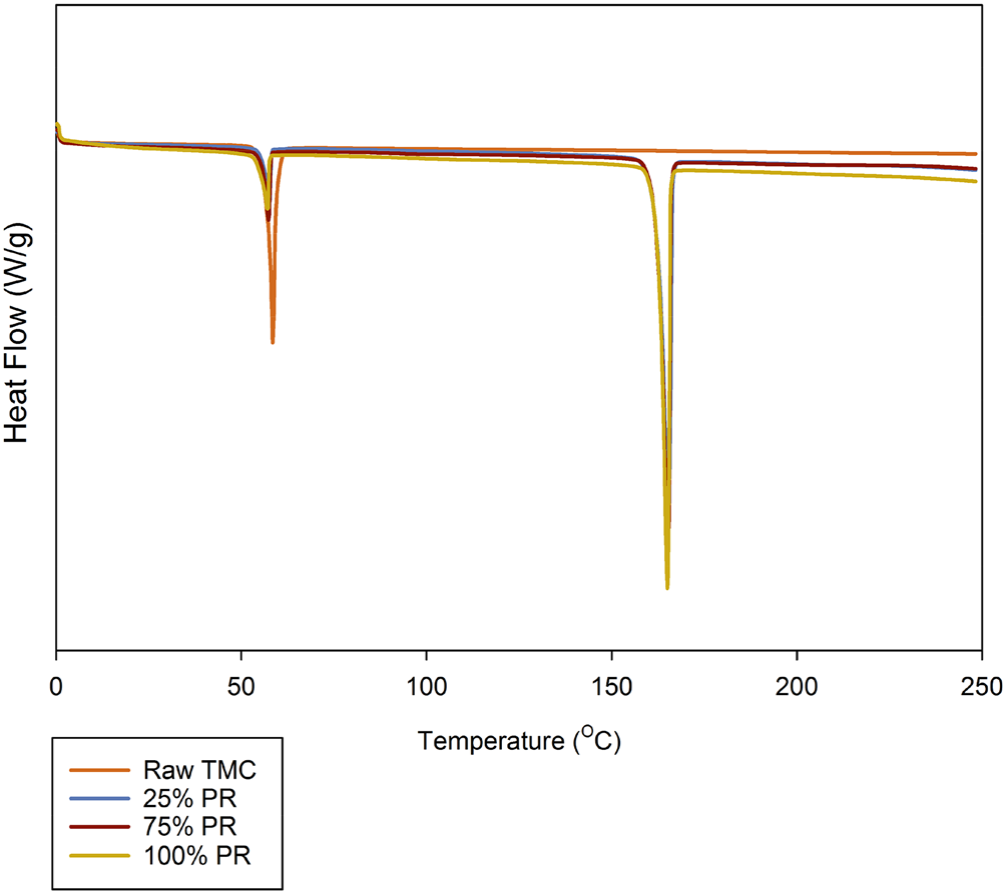
Representative differential scanning calorimetry (DSC) thermograms of Raw TMC and co-SD TMC: Man particles.

Figure 5 shows representative thermograms of raw TMC and co-SD TMC: Man particles with the corresponding thermodynamic properties in Table 3. Raw TMC had a single endothermic transition from the solid-to-liquid state at 58 °C which is the main phase transition temperature and melting temperature (T_m_) of TMC. On the other hand, the co-SD TMC: Man particles at all the three pump rates showed two endothermic transitions around 57 °C and 164 °C. The first transition belongs to TMC, while the second transition is that of Man as previously observed^45^.

**Table 3:** DSC thermal analysis (n = 3, mean ± standard deviation).

Powder composition (molar ratio)	Spray drying pump rate (%)	T_peak_ (°C)	ΔH (W/g)
Raw TMC	N/A	58.15 ± 0.38	90.65 ± 7.37
Co-SD TMC:Man 25:75	25	57.9 ± 0.01	21.79 ± 0.09
		164.71 ± 0.31	186.93 ± 2.02
Co-SD TMC:Man 25:75	75	57.65 ± 0.03	26.41 ± 2.43
		164.62 ± 0.18	185.37 ± 12.68
Co-SD TMC:Man 25:75	100	57.42 ± 0.06	20.18 ± 3.00
		164.52 ± 0.02	156.97 ± 19.85

### Attenuated total reflectance (ATR)–Fourier transform infrared (FTIR) spectroscopy

**Figure 6:**
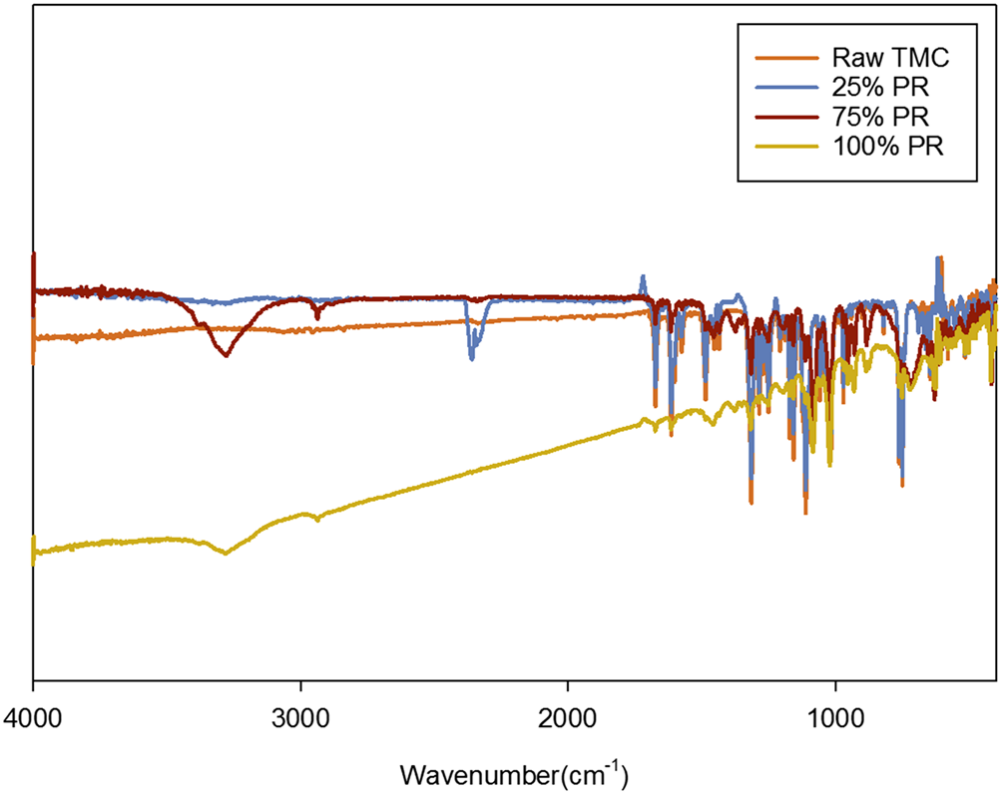
Attenuated total reflectance–Fourier transform infrared (ATR–FTIR) spectra of Raw TMC and co-SD TMC: Man particles.

For molecular fingerprinting in a non-invasive and non-destructive manner, ATR-FTIR is used. ATR-FTIR spectra of raw TMC and all co-SD TMC: Man particles showed similar absorption bands in the fingerprint region, as seen in Fig. 6. The distinctive peaks are seen at 749 cm^−1^ (CH-out of plane deformation in ortho-disubstituted benzene), 1615 cm^−1^ (benzene ring stretch seen in aromatic compounds), and 1314 cm^−1^ (CF_3_ antisym stretch seen in CF_3_ attached benzene ring) serve to be the characteristic bands of TMC molecule. These bands are also seen in the co-SD formulations in addition to the band seen around 3200 cm^−1^ in 75% and 100% PR particles. This is suggestive of H-bonding between TMC and Man particles.

### Confocal Raman microspectroscopy (CRM), chemical imaging, and mapping

**Figure 7:**
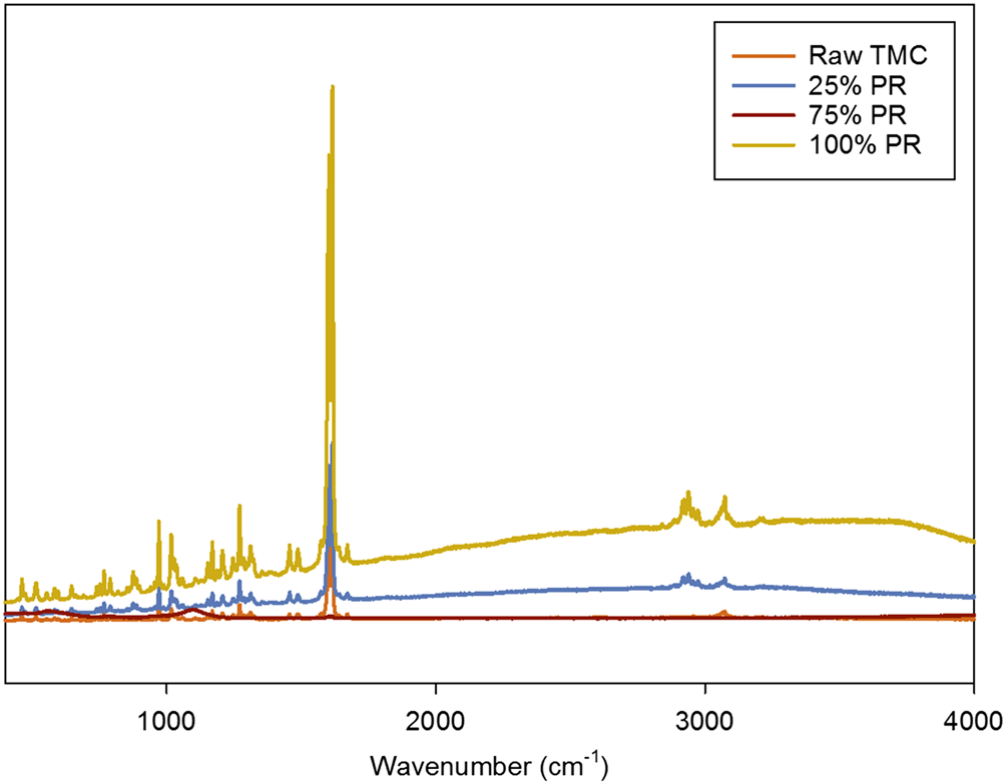
Raman spectra of Raw TMC and co-SD TMC: Man particles.

For molecular fingerprinting in a non-invasive and non-destructive manner, Raman spectroscopy is used and complements ATR-FTIR. The Raman spectra obtained using a 512 nm laser is seen in Fig. 7. The spectra obtained compliment the absorption bands seen in ATR-FTIR. Moreover, the Raman spectra of raw TMC and the co-SD TMC: Man particles at all pump rates look similar to each other suggesting that the co-SD formulations of TMC: Man at all pump rates didn’t affect the intermolecular interaction of the two compounds.

### In vitro aerosol dispersion performance

**Figure 8:**
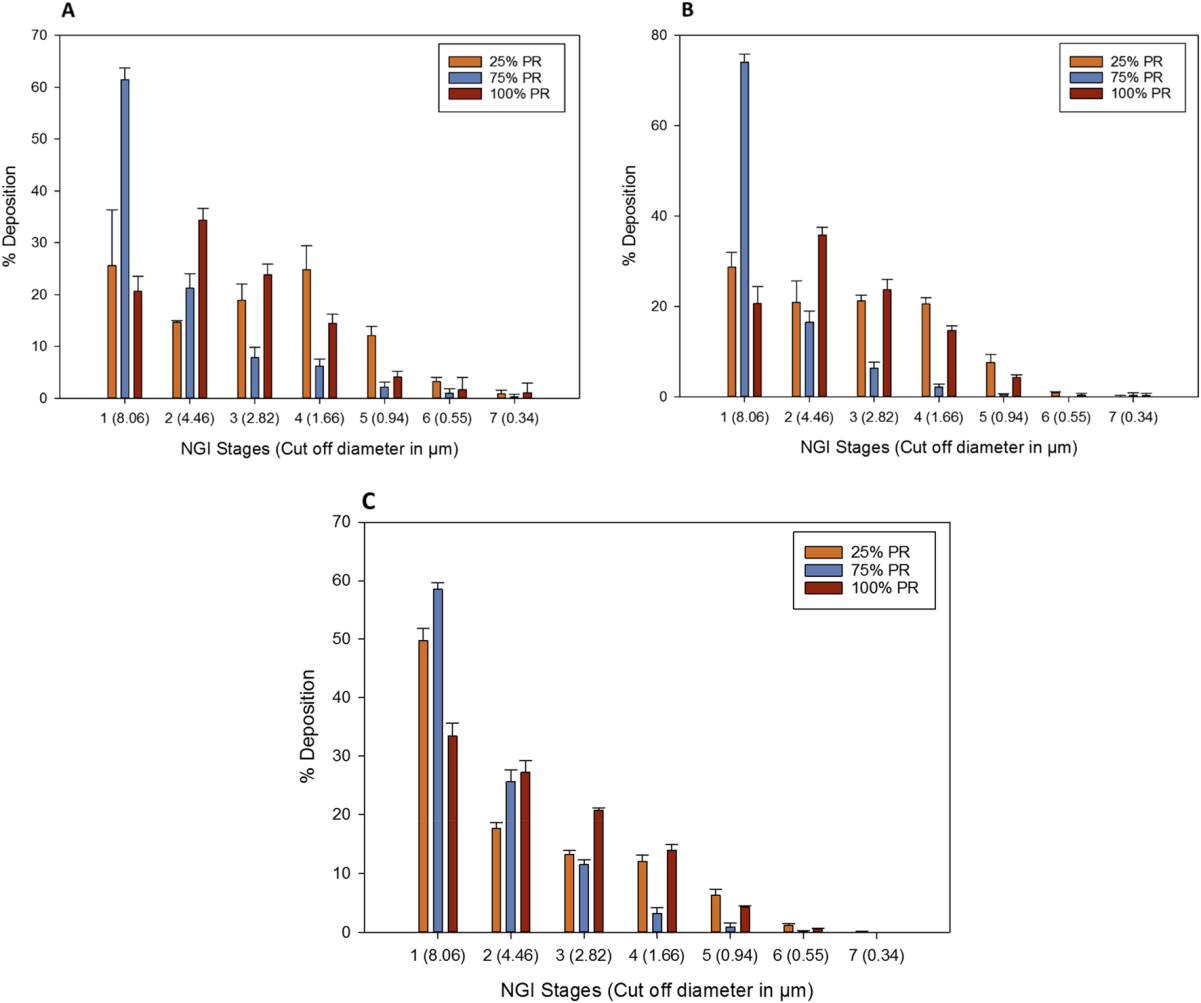
In vitro aerosol dispersion performance of co-SD TMC: Man particles as DPIs using the NGI and the FDA-approved human DPI devices (**A**) Aerolizer; (**B**) Neohaler; and (**C**) HandiHaler.

The in vitro aerosol dispersion performance of co-SD TMC: Man particles are shown in Fig. 8 and Table 4. Figure 8 shows the aerosol deposition on individual stages of the NGI when the three different human DPI devices were used. Table 4 lists the ED, FPF, RF, calculated MMAD, and GSD of the co-SD TMC: Man particles at the three pump rates. It can be seen that the highest ED was obtained with the Aerolizer and Neohaler DPI devices. The ED provided with the HandiHaler device was very high in the range of 91–98%. The highest FPF of 38.75 ± 2.33 obtained was with 100% PR particles with the HandiHaler DPI device followed by the same particles aerosolized with Neohaler DPI device giving an FPF of 36.12 ± 1.67. The smallest MMAD (a calculated value) of 3.56 ± 0.51 µm was obtained with 25% PR particles when used with Aerolizer device followed by 4.56 ± 0.56 µm of 25% PR particles with Neohaler device. The calculated MMAD of Co-SD TMC: Man particles at 100% PR was in the range of 5 -5.5 µm using all the inhaler devices.

**Table 4:** In vitro aerosol dispersion performance of co-SD TMC: Man as DPIs (n = 3, mean ± standard deviation).

Inhaler device	Aerolizer		
SD pump rate	25%	75%	100%
ED (%)	96.36 ± 0.78	100.42 ± 2.17	101.68 ± 2.07
FPF (%)	28.20 ± 4.31	5.61 ± 4.05	26.28 ± 3.64
RF (%)	74.38 ± 33.08	38.57 ± 16.78	79.40 ± 34.44
MMAD (µm)	3.56 ± 0.51	11.74 ± 0.70	5.31 ± 0.51
GSD	2.15 ± 0.10	3.00 ± 0.62	2.61 ± 1.03

Inhaler device	Neohaler		
SD pump rate	25%	75%	100%
ED (%)	102.03 ± 0.45	89.56 ± 13.30	100.18 ± 1.98
FPF (%)	32.63 ± 3.82	7.32 ± 1.28	36.12 ± 1.67
RF (%)	71.30 ± 30.96	25.99 ± 11.32	79.38 ± 34.48
MMAD (µm)	4.56 ± 0.56	14.99 ± 1.96	5.07 ± 0.26
GSD	1.87 ± 0.24	2.49 ± 0.26	1.95 ± 0.07

Inhaler device	HandiHaler		
SD pump rate	25%	75%	100%
ED (%)	91.43 ± 9.26	97.69 ± 0.79	92.53 ± 2.70
FPF (%)	28.54 ± 1.51	15.57 ± 0.62	38.75 ± 2.33
RF (%)	50.27 ± 21.82	41.43 ± 17.96	66.61 ± 28.88
MMAD (µm)	6.93 ± 0.63	9.44 ± 0.14	5.47 ± 0.10
GSD	2.69 ± 0.08	2.03 ± 0.02	2.08 ± 0.03

*ED* emitted dose, *FPF* fine particle fraction, *RF* respirable fraction, *MMAD* mass median aerodynamic diameter, *GSD* geometric standard deviation.

### In vitro transepithelial electrical resistance analysis (TEER)

**Figure 9:**
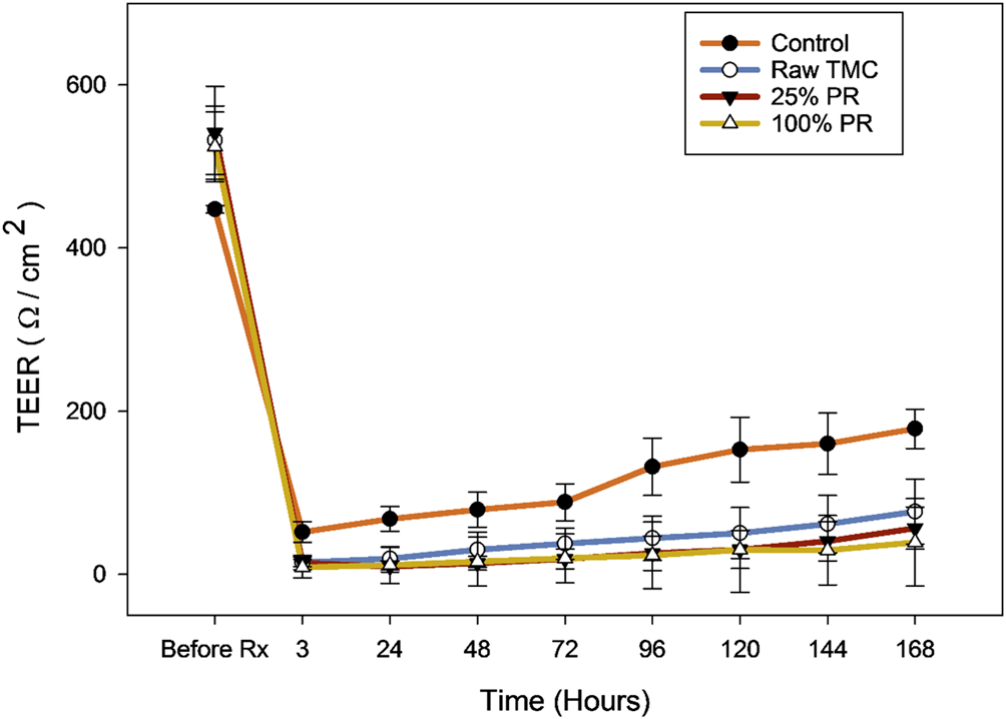
In vitro transepithelial electrical resistance (TEER) analysis of Calu-3 lung epithelial cells exposed to representative formulations in air-interface culture (AIC) conditions using Penn-Century Microsprayer Aerosolizer.

The TEER values for the raw TMC and co-SD TMC: Man particles are displayed in Fig. 9. The control value represents the cells treated with the vehicle in which the test formulations were dissolved, in this case, 10% ethanol + 90% EMEM cell media. The raw TMC and representative co-SD TMC: Man particles had decreased TEER value right after treatment which gradually recovered. It can be seen that the control cells recovered faster than the drug-treated cells.

## Discussion

This is the first study to report on DPI formulations of TMC for targeted pulmonary inhalation aerosol drug delivery. In addition, this is the first study to report on the physicochemical solid-state characteristics of raw TMC. This study has identified mannitol to be a suitable excipient to formulate TMC into small and spherical solid-state particles in the size range of a few hundred nanometers to a few micrometers. The particle size presented in this study is in the desired range for delivery of particles to the lower airways, as predicted by the in vitro aerosol dispersion and deposition on the lower stages (i.e. smaller aerodynamic size) of the inertial impactor. Advanced spray drying in closed-mode under the reported conditions (Table 1) was successfully used with methanol as the solvent to design and produce engineered solid-state particles with physical properties necessary for dry powder aerosol formulation. Initially, TMC was spray dried individually using methanol and isopropanol at various concentrations using different spray drying conditions. Despite the change in concentrations and solvent, TMC particles were not formed. A similar observation was made when another Nrf2-activator drug dimethyl fumarate (DMF) was spray dried, as reported earlier^20^. In that study^20^, mannitol was used to promote particle formation by hydrogen-bonding with DMF. Hence, in this study, a similar approach was adopted and Man was used as an excipient to successful form inhalable TMC nanoparticles and microparticles in the solid-state. Several different molar ratios of TMC and Man were tested to form co-SD particles at different advanced spray drying conditions. The three selective formulations were chosen for characterization and development based on the preliminary aerosol performance and the higher particle yield during spray drying.

From the SEM images (Fig. 2), it can be seen that the co-SD TMC: Man particles formed small, spherical and smooth particles at 25% and 100% PR. The 75% PR particles were relatively larger with some degree of particle coalescence happening, nonetheless, the discrete particles that were measurable were small with the slightly corrugated surface. The particle coalescence observed could also be due to the smearing on to the SEM stub for imaging purpose. This could have caused the primary particles to break or sinter. In any case, co-SD particles were sized and the average particles size and size distribution were calculated. The co-SD TMC: Man particles at 25% and 100% PR had relatively smaller particles compared to the 75% PR. The span value which is an indication of the size distribution width, in other words, this value suggests how far is the 90% and 10% percentile particles from the median diameter. In this study, the span values of 0.63, 1.27 & 0.89 indicating that the particle size distribution is narrow, which correlates well with the SEM images.

Thermal analysis by DSC identified the main phase transition of melting (T_m_) for the raw TMC at ~ 58 °C and the change in enthalpy (ΔH) was calculated to be ~ 90 kJ/mol (Table 3 and Fig. 5). This first-order phase transition temperature agrees well with the T_m_ of the parent chalcone which is reported to be 55–57 °C in ChemSpider and PubChem. This the only transition observed during the thermal analysis, further indicating that the compound was crystalline and in excellent agreement with the XRPD data. The co-SD TMC: Man particles at all the three pump rates also exhibited the same transition around 57–58 °C, which indicates the presence of the same polymorph. This was further confirmed using XRPD diffractogram peaks (Fig. 4) of raw and co-SD TMC: Man. All the prominent peaks of raw TMC seen at 12.8, 13.5, 14.9, 16.1, 17.1, 18.3, 22.0, 23.0, 24.9, 25.7, 26.9 and 28.4 2θ degrees were also seen in the co-SD TMC: Man particles at all pump rates further confirming the that TMC exists in the same crystalline form before and after spray drying. In other words, there was no polymorphic interconversion seen as evident from both XRPD and DSC results. There is no previous report on the solid-state properties of TMC, as this is the first time the XRPD diffractograms and DSC thermograms of TMC are reported.

The elemental atomic analysis using SEM–EDX (Fig. 3) provides confirmation of the chemical presence of the fluorine atom at 0.68 keV which is from the CF_3_ group attached to the benzene ring. The gold atoms seen in the spectra are the M-lines from the gold coating and do not represent the molecular composition of the powder formulation. In excellent agreement with the SEM–EDX data (Fig. 3) is the molecular fingerprinting spectroscopy data. The vibrational spectroscopy data using ATR-FTIR (Fig. 6) and Raman (Fig. 7) also show the peaks of CF_3._ The peaks seen in the region between 700–1700 cm^−1^ wavenumbers match in both raw TMC and co-SD samples, which serves as a molecular fingerprint identification of TMC in the pure solid-state and in the co-SD powder formulations. However, there were additional peaks around 3200 cm^−1^ seen in some co-SD TMC: Man powders suggesting H-bonding between the TMC and mannitol molecules which agrees well with their chemical structures.

From the in vitro TEER results in air-interface culture (AIC) conditions (Fig. 9), it can be seen that the co-SD TMC: Man formulations upon aerosolization directly onto the cell temporarily disrupted the monolayer of the Calu-3 cells in the transwell inserts for a brief period of time. The TEER of this human lung bronchial cell line recovered within hours. The recovery of drug-treated cells was slower than that of the control cells which was treated with aerosolized cell culture media.

The NGI stage deposition profiles seen in Fig. 8 using Aerolizer and Neohaler showed that the 75% PR particles had a higher deposition on Stage 1 compared to the lower stages, while 25% and 100% PR particles had less deposition on Stage 1. However, with HandiHaler all the three co-SD powders had a higher deposition on Stage 1. From Fig. 8, it is evident that the co-SD TMC: Man 75% PR powder deposition profile was considerably different from that of the other profiles by two PR powders. Even though the 25% and 100% PR powders NGI deposition profile look similar, there was a statistically significant difference between the deposition profiles when Neohaler and HandiHaler DPI devices were used, as reflected in the aerosol performance parameters (Table 4). On the other hand, for 25% PR and 100% PR powders, there was no significant difference between Aerolizer and Neohaler but 75% PR showed no statistical difference between the Aerolizer and HandiHaler devices (Table 4).

From the particle sizing data (Table 2), it was seen that the geometric diameters were few hundred nanometers to a few micrometers, and all less than 3 µm in linear geometric diameter. Yet, the MMAD calculated from the NGI results was in the range of 3.5–15 µm (Table 4). This can be due to the physical properties influencing the aerodynamic properties^46^. Firstly, nanoparticles^47,48^ have an increased surface area-to-volume ratio leading to higher surface forces that could cause increased interparticulate interactions leading to cohesive forces between the solid-state particles. On the other hand, the shear stress generated from the DPI devices could have been insufficient to completely disperse all of the particle aggregates into their primary particles. The co-SD TMC: Man particles at 25% PR, which had a geometric particles size range of 520 nm-980 nm (Table 2) was better dispersed by the lower resistance (low shear stress) DPI device, the Aerolizer (Fig. 8 and Table 4), whereas, the 75% PR particles had a geometric size range of 790 nm–2.26 µm (Table 2).

**Figure 10:**
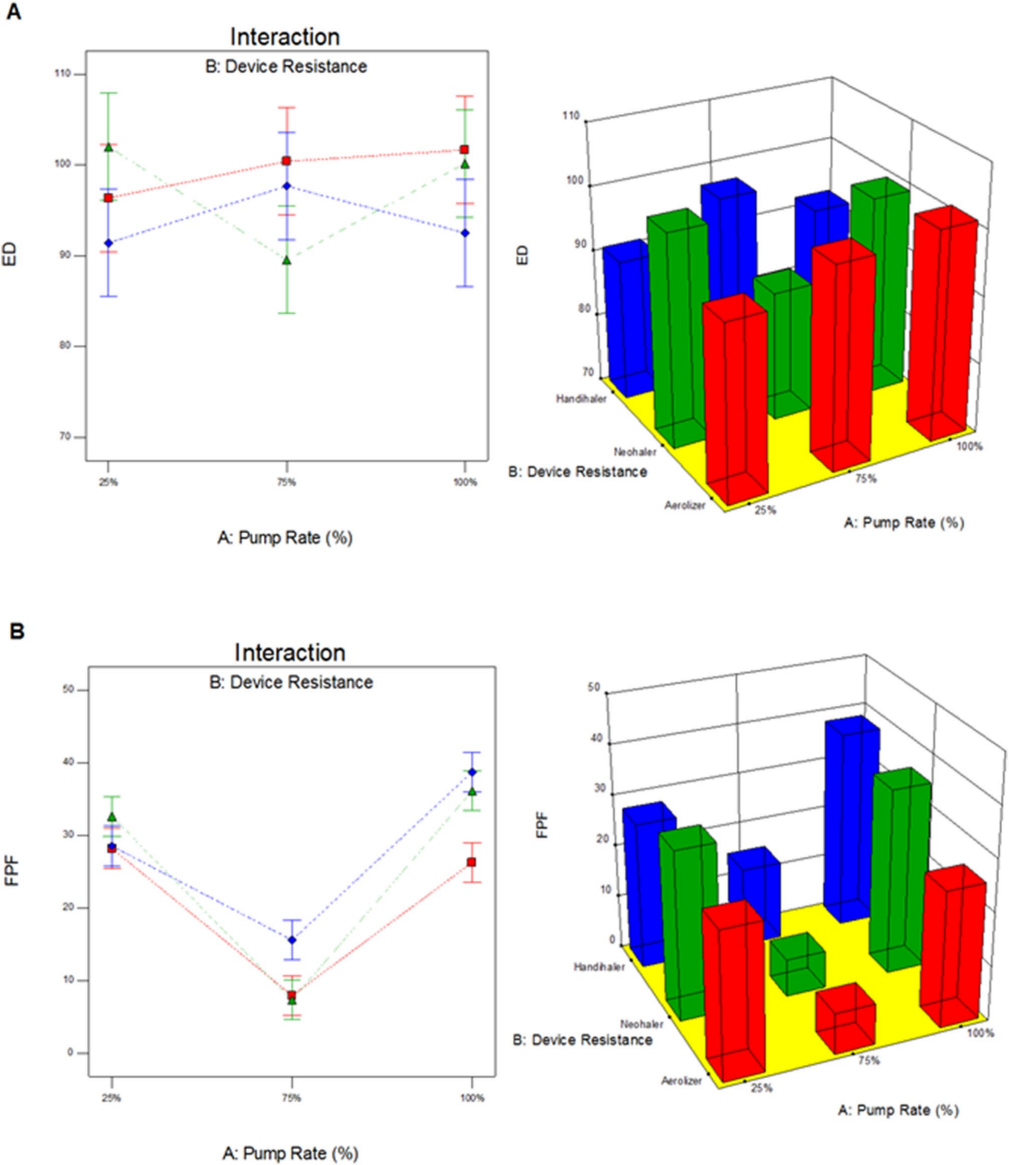

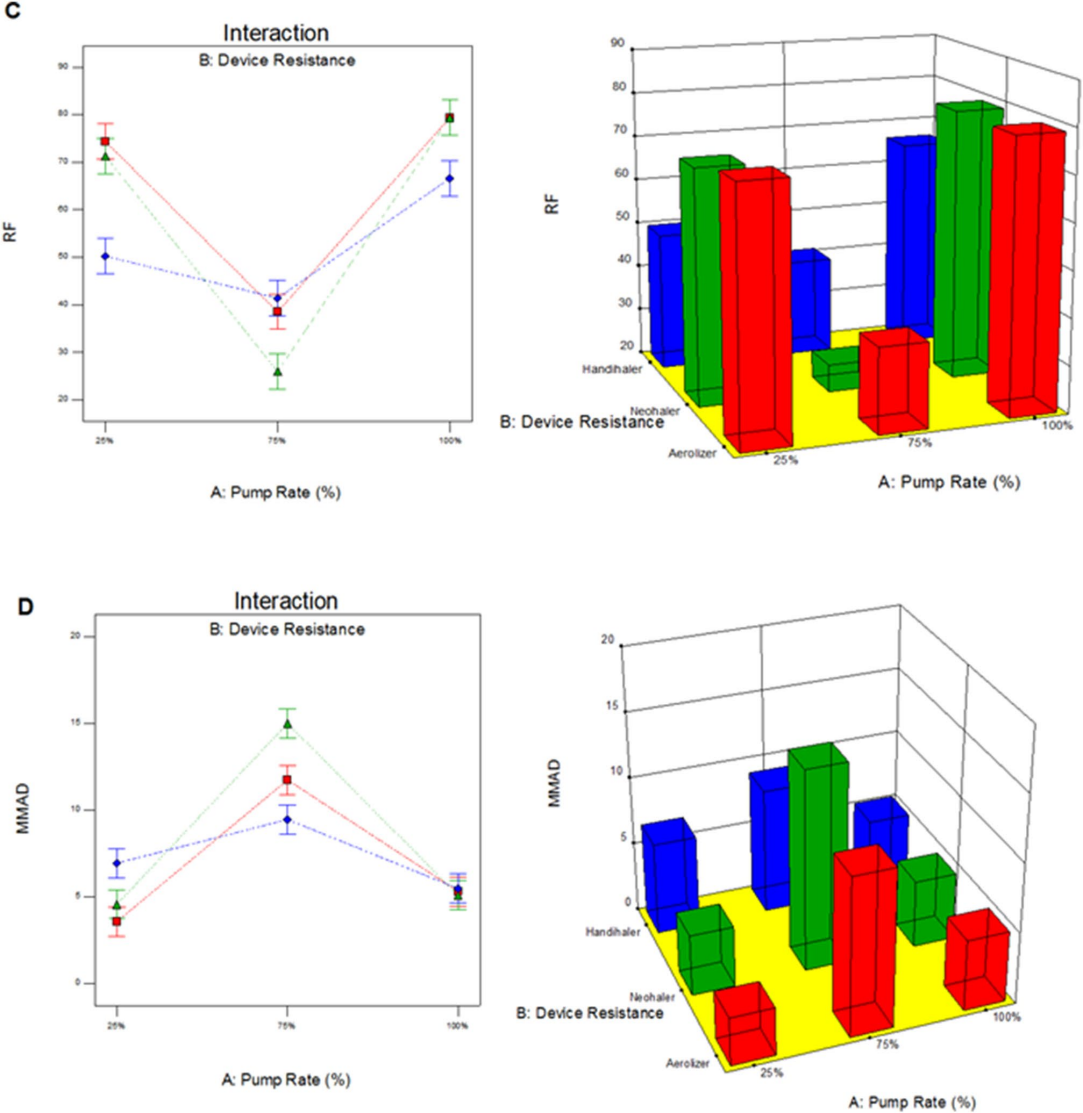
Process parameter interaction graphs showing the influence of spray drying pump rate and inhaler device resistance on in vitro aerosol dispersion performance for co-SD TMC Man powder as DPIs for (**A**) ED; (**B**) FPF; (**C**) RF; and (**D**) MMAD.

The interaction correlation between spray drying inlet feed pump rate and the inhaler device’s inherent specific resistance was analyzed using Design-Expert software (Stat-Ease, Minneapolis, MN). Figure 10 shows the 3-D surface and interaction plots where ED, FPF, RF, and MMAD were considered as design response terms. The ED (p-value = 0.2647) was not a significant term. Evidently, neither the pump rate nor the device resistance affected the fraction of powder emitted from the inhaler device. The overlapping error bars in the interaction plot in Fig. 10A demonstrates the same. However, the opposite trend was correlated for FPF (Fig. 10B), RF (Fig. 10C), and MMAD (Fig. 10D) (p < 0.0001), which were affected by both pump rate and device resistance. From Fig. 10, it can be seen that co-SD TMC: Man 75% PR particles had the lowest FPF, RF and highest MMAD. However, 25% and 100% PR particles had comparable results for FPF, RF and MMAD. At lower (25%) and higher (100%) spray drying pump rates TMC: Man formed relatively smaller and spherical particles that dispersed well with low and low-medium resistance inhaler devices. The mediocre performance of the 75% spray drying pump rate particles can be related to the physicochemical properties of the powder.

## Conclusions

This systematic and comprehensive study reports for the first time on innovative dry powder inhaler formulations of the novel synthetic trifluorinated Nrf2-agonist TMC molecularly mixed with a d-mannitol in the solid-state. This study is the first to report on the comprehensive characterization of the physicochemical properties of TMC including T_m_, crystalline phase, and spectroscopy molecular fingerprinting by both ATR-FTIR and Raman spectroscopy. The study also successfully designed, produced, and optimized inhalable TMC nanoparticles and microparticles in the solid-state molecularly mixed with d-mannitol as an excipient engineered advanced organic closed-mode spray drying technique at three rationally chosen pump rates. All of these microparticulate/nanoparticulate powders possessed the essential particle properties necessary for dry powder aerosolization and all readily aerosolized with three different human DPI devices that vary in resistance and shear stress. The correlation between spray drying process parameters, inhaler device resistance (shear stress), and aerosol properties were correlated in a meaningful manner.

## Materials and methods

### Materials

Synthetic TMC (2-trifluoromethyl-2′-methoxychalone) with a molecular formula of C_17_H_13_F_3_O_2_ and molecular weight (MW) of 306.28 g/mol was generously provided by Cureveda LLC. d-mannitol (C_6_H_14_O_6_; MW: 182.17 g/mol) was obtained from Acros Organics (New Jersey, USA). Methanol (HPLC grade, ACS-certified grade, purity 99.9%) was obtained from Fisher Scientific (Fair Lawn, New Jersey). Hydranal-Coulomat AD was obtained from Sigma-Aldrich. The nitrogen gas used was ultra-high purity (UHP) nitrogen gas (Cryogenics and Gas facility, The University of Arizona, Tucson, AZ). Raw TMC was stored in sealed glass desiccators over Indicating Drierite desiccant at − 80 °C. Raw d-mannitol was used as received and stored under room conditions. Other chemicals were stored under room conditions.

### Methods

#### Preparation of co-SD particles by organic solution advanced spray drying in closed-mode

Organic solution advanced spray drying process in the absence of water was performed in a closed-mode using a Büchi B-290 Mini spray dryer with a high-performance cyclone in a closed mode using UHP dry nitrogen gas as the atomizing drying gas. The feed solution consists of drug dissolved in methanol. For the two-component system, the components were dissolved successively in the solvent consisting of the drug with Man in rationally selected molar ratios. Table 1 lists the spray drying conditions used for the dry powder aerosol formulation development. The drying gas atomization rate (670 L/h at 35 mmHg) and aspiration rate (35 m^3^/h at 100% rate) were maintained constant during all the experiments. Three feed pump rates were employed to obtain particles using pump rates of 7.5 mL/min (low, 25%), 22.5 mL/min (medium–high, 75%), and 30 mL/min (high, 100%). The stainless steel two-fluid nozzle used to spray the feed solution had a tip diameter of 0.7 mm with 1.5 mm gas cap. All co-SD powders were carefully stored in sealed glass vials stored in sealed glass desiccators over indicating Drierite desiccant at − 20 °C under ambient pressure.

#### Scanning electron microscopy (SEM) and energy dispersive X-ray (EDX) spectroscopy

Using conditions similar to those previously reported^20,45,49–51^, raw and spray dried particles were visualized using scanning electron microscopy (SEM). The powder samples were sputter coated with gold for 90 s under Argon plasma. An electron beam of 30 kV voltage was used for imaging.

#### Particle sizing and size distribution image analysis using SEM micrographs

The mean particle size, standard deviation, and particle size range were determined digitally using SigmaScan Pro 5 (Systat software, San Jose, CA) using similar conditions that have been previously reported^20,52^. Representative SEM micrographs of each powder sample at 10,000 × magnification were analyzed by measuring the diameter of at least 100 particles.

#### X-ray powder diffraction (XRPD)

Using conditions similar to previously reported^20,45,49–51^, X-ray powder diffraction (XRPD) patterns were collected at room temperature using copper Kα radiation (45 kV, 40 mA, and λ = 1.5406 Å) between 5.0° and 70.0° (2θ) for raw TMC and 5.0° and 50.0° (2θ) for co-SD powders at a scan rate of 2.00°/min at ambient temperature. A zero background silicon wafer sample holder was used.

#### Differential scanning calorimetry (DSC)

Using conditions similar to previously reported^20,45,49–51^, thermal analysis and phase transition measurements were performed. Approximately 1–10 mg of sample was placed into an anodized aluminum hermetic DSC pan that was sealed with the T-Zero hermetic press. The samples were heated from 0.00 to 250.00 °C at a scanning rate of 5.00 °C/min.

#### Confocal Raman microspectroscopy (CRM), and chemical imaging

Confocal Raman microspectroscopy (CRM) provides noninvasive and nondestructive microspectroscopic component analysis of DPI formulations. Using similar conditions previously reported^20,45,49–51^, Raman spectra were obtained at 514 nm laser excitation using Renishaw InVia Reflex using a 20 × magnification objective on a Leica DM2700 optical microscope. Raman spectral map was obtained with the stage moved in increments of 20 × 20 µm in each axis. Each map point was acquired 1 accumulation using 2 s of detector exposure time per accumulation.

#### Attenuated total reflectance (ATR)–Fourier-transform infrared (FTIR) spectroscopy

Each spectrum was collected for 32 scans at a spectral resolution of 8 cm^−1^ over the wavenumber range of 4000–400 cm^−1^. A background spectrum was subtracted from each sample spectrum. These conditions are similar to those in our previous reports^45,49–51^.

#### In vitro aerosol dispersion performance

In vitro aerosol dispersion analysis was conducted in accordance with USP Chapter $\left\langle {601} \right\rangle$ specifications^53^ on aerosols and using conditions similar to previously reported^45,49–51^ using the Next Generation Impactor (NGI) with a stainless steel induction port (USP throat) attachment. Three FDA approved human DPI devices Aerolizer (Merck, Germany), Neohaler (Novartis, Switzerland) and HandiHaler (Boehringer Ingelheim, Germany) were used for the aerosol dispersion. An airflow rate (Q) of 60 L/min (adult airflow rate) was adjusted and measured before each experiment. The mass of powder deposited on each stage was quantified gravimetrically. Quali-V clear HPMC size 3 inhalation grade capsules were used with about 10 mg of powder filled in it. Three capsules were used in each experiment. In vitro aerosolization was evaluated in triplicate (n = 3) under ambient conditions. Design of experiments (DoEs) was conducted using Design-Expert 8.0.7.1 software (Stat-Ease Corporation, Minneapolis, MN). A full-factorial design of 3^2^ for co-SD TMC: Man systems was utilized.

The emitted dose (ED), fine particle fraction (FPF) and respirable fraction (RF) were calculated similar to previous reports^45,49–51^. The ED was determined as the difference between the initial mass of powder loaded in the capsules and the remaining mass of powder in the capsules following aerosolization. The ED (%) Eq. (1) was used to express the percentage of ED based on the total dose (TD) used. The fine particle dose (FPD) was defined as the dose deposited on stages 2 to 7. The FPF % in Eq. (2) was expressed as the percentage of FPD to ED. The RF % in Eq. (3) was used as the percentage of FPD to total deposited dose (DD) on all NGI stages.

1 $$ Emitted \;dose\; fraction\; (ED\% ) = \frac{ED}{{TD}} \times 100\% $$

2 $$ Fine \;particle\;fraction \;(FPF\% ) = \frac{FPD}{{ED}} \times 100\% $$

3 $$ Respirable \;fraction\; (RF\% ) = \frac{FPD}{{DD}} \times 100 \% $$

#### In vitro transepithelial electrical resistance analysis in air-interface culture (AIC)

Calu-3 human lung adenocarcinoma cell line derived from the bronchial submucosal airway region was grown in Eagle’s minimum essential medium (EMEM), 10% (v/v) fetal bovine serum (FBS), Pen-Strep (100 U/mL penicillin, 100 µg/mL), Fungizone (0.5 µg/mL amphotericin B, 0.41 µg/mL sodium deoxycholate) as previously reported ^54–56^. They were seeded at a concentration of 500,000 cells/mL in Costar Transwells^®^ (0.4 μm polyester membrane, 12 mm for a 12-well plate). After few days of growth, when the cells reached a TEER value of about 1000 Ω/cm^2^ the media was removed apical side to facilitate air-interface culture (AIC) conditions. When the TEER values reached approximately 500 Ω/cm^2^ (indicating a confluent monolayer at AIC conditions), the cells were exposed to 100 µM of the test formulation dissolved in non-supplemented EMEM media. The aerosol formulations were delivered as a liquid aerosol using the Penn-Century MicroSprayer Aerosolizer Model IA-1B. TEER values were then recorded for up to 7 days after aerosol treatment, as previously reported^53,56^. Media was added to the apical side temporary for TEER measurements, after which the cells returned to AIC conditions.

### Statistical analysis

Design of experiments (DoEs) for in vitro aerosol performance was conducted using Design-Expert 8.0.7.1 software (Stat-Ease Corporation, Minneapolis, MN). A full-factorial design of 3^2^ for co-SD TMC: Man systems was utilized for in vitro aerosol dispersion performance testing. Aerosol dispersion parameters and process parameters interactions were evaluated using the Analysis of Variance (ANOVA) test performed using SigmaPlot Version 13 (Systat software, Inc, San Jose, CA) and Design-Expert software programs. All experiments were performed in at least triplicate (*n* = 3).
